# Components of the Female Sex Pheromone of the Newly-Described Canola Flower Midge, *Contarinia brassicola*

**DOI:** 10.1007/s10886-022-01369-z

**Published:** 2022-06-30

**Authors:** Daniel P. Bray, David R. Hall, Steven J. Harte, Dudley I. Farman, Meghan A. Vankosky, Boyd A. Mori

**Affiliations:** 1 Natural Resources Institute, University of Greenwich, Chatham Maritime, Kent, UK; 2 Agriculture and Agri-Food Canada, Saskatoon, Canada; 3 Department of Agricultural, Food and Nutritional Science, University of Alberta, Edmonton, Canada

**Keywords:** Canola flower midge, Cecidomyiidae, Oilseed rape, *Brassica*, 2-Acetoxynonane, 2,7-Diacetoxynonane, Stereochemistry

## Abstract

The canola flower midge, *Contarinia brassicola* Sinclair (Diptera: Cecidomyiidae), is a newly-described species that induces galls on canola, *Brassica napus* Linnaeus and *Brassica rapa* Linnaeus (Brassicaceae). Identification of the sex pheromone of *C. brassicola* is essential to developing monitoring tools to elucidate the geographic range and hosts of this new pest, and the extent to which it threatens the $30 billion Canadian canola industry. The aim of this study was to identify and synthesize the female-produced sex pheromone of *C. brassicola* and demonstrate its effectiveness in attracting males to traps in the field. Two peaks were identified through GC-EAG analysis of female-produced volatiles which elicited electrophysiological responses in male antennae. These peaks were initially characterized through GC–MS and synthesis as 2,7-diacetoxynonane (major component) and 2-acetoxynonane (minor component), and the racemic compounds elicited EAG responses in male antennae. All four stereoisomers of 2,7-diacetoxynonane were synthesized and the naturally-produced compound was shown to be primarily the (2*R,*7*S*)-isomer by analysis on an enantioselective GC column, with a small amount of (2*R,*7*R*)-2,7-diacetoxynonane also present. The configuration of the minor component could not be determined because of the small amount present, but this was assumed to be (2*R*)-2-acetoxynonane by comparison with the configuration of the other two components. In field trials, none of the four stereoisomers of 2,7-diacetoxynonane, presented individually or as a racemic mixture, was attractive to male *C. brassicola*. However, dispensers loaded with a 10 µg:1 µg blend of (2*R,*7*S*)- and (2*R,*7*R*)-2,7-diacetoxynonane caught large numbers of male *C. brassicola* and significantly more than other blends tested. The addition of 0.5 µg of (2*R*)-2-acetoxynonane to this blend further increased the number of males caught. In future work, we will seek to identify the optimum trapping protocol for the application of the pheromone in monitoring and surveillance.

**Supplementary Information:**

The online version contains supplementary material available at 10.1007/s10886-022-01369-z.

## Introduction

The canola flower midge, *Contarinia brassicola* Sinclair (Diptera: Cecidomyiidae), induces galls on canola, *Brassica napus* Linnaeus and *Brassica rapa* Linnaeus (Brassicaceae) (Mori et al. 2019). The galls were first discovered in the province of Saskatchewan in 2012 and originally attributed to the swede midge, *Contarinia nasturtii* (Kieffer) (Diptera: Cecidomyiidae), the only other species of cecidomyiid known to infest canola in North America (Mori et al. 2019). However, adults of the two species exhibited differences in the morphology of the wings, antennae, and genitalia, as well as the shape of flower galls produced. Results of phylogenetic analyses also supported *C. brassicola* as a distinct species within the genus *Contarinia* (Mori et al. 2019). *Contarinia brassicola* appears to be the main species of *Contarinia* on canola across the Canadian prairies, where *C. nasturtii* has not been detected since 2007 (Mori et al. 2019; Vankosky et al. 2022). The origin of *C. brassicola*, and its importance as a pest species threatening the $30 billion Canadian canola industry (Canola Council of Canada 2021), remain to be determined.

*Contarinia brassicola* adults begin to emerge in June and July on the Canadian prairies, which coincides with bud formation and early flowering in canola (Mori et al. 2019; Vankosky et al. 2022). Females lay eggs on developing canola buds, and, after hatching, larvae feed cryptically within the flower bud resulting in gall formation. These galls prevent pod formation and result in yield loss. Mature larvae leave the galled flowers and form cocoons in the soil. A portion of the larvae pupate and emerge as a second generation, while others appear to undergo diapause for emergence the following year (Campbell et al. 2020; Vankosky et al. 2022). To date, *C. brassicola* has only been found in North America (Campbell et al. 2020; Mori et al. 2019).

Adult females of at least 19 species of Cecidomyiidae have been demonstrated to produce sex pheromones attractive to males (Hall et al. 2012; Xu et al. 2020). As adult males and females may only live for 1–2 days, these chemical signals are likely to be crucial to successful mate finding and reproduction (Hall et al. 2012). Whether female *C. brassicola* produce a sex pheromone attractive to males is currently unknown. Male *C. nasturtii* possess specialized structures, *sensilla circumfila*, on the antenna, which contain olfactory neurons sensitive to female-produced pheromones (Boddum et al. 2010). Male *C. brassicola* possess similar structures, not present on females (Mori et al. 2019), which may indicate the presence of a sex-specific pheromone in this species. Results of trials in emergence cages indicated that *C. brassicola* is not attracted to traps baited with synthetic *C. nasturtii* pheromone (Mori et al. 2019).

Traps baited with lures containing synthetic versions of female-produced sex pheromones are available for monitoring a number of species of cecidomyiids (Hall et al. 2012) including the congeneric pests, swede midge, C*. nasturtii* (Boddum et al. 2009) and pea midge, *C. pisi* (Winnertz) (Hillbur et al. 1999, 2000, 2001). A suitable pheromone-baited trap could be used to determine the importance of *C. brassicola* as a crop pest, and to ascertain the geographical range and plant hosts of this species.

The aims of this study were to determine whether female *C. brassicola* produce a pheromone, and whether a synthetic version of the pheromone can be used to attract male *C. brassicola* to traps in the field. Headspace entrainments were collected from groups of newly-emerged males and females, and a combination of GC–MS and GC-EAG analyses was applied to identify potential pheromone components detected by male antennae. Field trials with synthetic pheromones were conducted in Canada to determine the blend of the pheromone components most attractive to male *C. brassicola*.

## Methods and Materials

### Collection and Rearing of Midges

*Contarina brassicola* larvae were collected from infested flowers of canola, *B. napus* L., throughout the growing season in 2017 and 2018 from several commercial canola fields near the towns of Melfort (52.862, -104.615) and Nipawin (53.364, -104.013) in northeastern Saskatchewan, Canada. Infested flowers were placed in petri dishes filled with a sterilized, damp, soil-less potting mix (Stringham 1971) and placed in a growth chamber (22 °C, 16:8 h D:L, ~ 70% RH). After 7–10 days, the soil-less potting mix was sieved, and cocoons containing pupae were collected. Pupae were sent to the UK under Department for Environment, Food & Rural Affairs, UK (DEFRA) import license and held in a licensed quarantine facility at the Natural Resources Institute (NRI), University of Greenwich, UK. Individual pupae were placed in sample cups with lids (2 ml; Kartell™ 2502, Fisher Scientific, UK) containing a piece of moist vermiculite to maintain humidity. The sex of emerging adults was determined according to antennal morphology under a dissecting microscope (× 400 magnification) (Mori et al. 2019).

### Collection of Volatiles from Virgin Midges

Volatiles were collected from virgin male and virgin female *C. brassicola* by placing single-sex groups into a silanized glass chamber with a glass frit at the upwind end (12 cm × 4 cm; Hamilton Laboratory Glass, Margate, Kent, UK). Insects were anesthetized briefly with CO_2_ to facilitate transfer from rearing vials to the glass containers. Air was drawn into the glass chambers at 200 ml/min through an activated charcoal filter (20 cm × 2 cm, 10–18 mesh; Fisher Scientific, Loughborough, UK) using a vacuum pump (DA7C; Charles Austen, West Byfleet, UK). Air flowed out of each chamber via a Pasteur pipette (4 mm i.d.) containing Porapak Q (200 mg, 50–80 mesh; Supelco, Gillingham, Dorset, UK) held between plugs of silanized glass wool. Porapak was cleaned through Soxhlet extraction with chloroform for 8 h and washed with dichloromethane prior to use. Dead midges were removed daily and replaced with newly-emerged individuals of the same sex. Most of the work was done with a collection from 68 females and one from 34 males made over 3 d, as these contained most material. Volatiles trapped on Porapak were eluted with 3 × 0.5 ml dichloromethane (Pesticide-Residue Grade) and stored at 4 °C until use.

### Analysis by Gas Chromatography Coupled to Electroantennographic Recording (GC-EAG)

GC-EAG analyses were carried out on a HP6890 GC (Agilent Technologies, Manchester, UK) fitted with flame ionization detector (FID) and fused-silica, capillary GC columns (30 m × 0.32 mm i.d. × 0.25 µm film thickness) coated with DBWax and DB5 (Supelco). Injections onto the DBWax column were in splitless mode (220 °C), carrier gas was helium (2.4 ml/min) and the oven temperature was held at 50 °C for 2 min and then programmed at 20 °C/min to 250 °C and held for 3 min. The effluents of the two columns were combined with a glass push-fit Y-tube connector (Agilent) connected to a second Y-tube connector with deactivated fused silica tubing (10 cm × 0.32 mm i.d.). One arm of this connector was connected with deactivated fused silica tubing (50 cm × 0.32 mm i.d.) to the FID (250 °C) and the other to an equal length of deactivated silica tubing passing through a heated transfer line (250 °C; Syntech, Hilversum, The Netherlands, now Kirchzarten, Germany) into a glass tube (4 mm i.d.) through which air passed (400 ml/min) over the EAG preparation.

EAG responses from male midges to female extracts and synthetic compounds were measured using a combined probe and amplifier (× 10) (INR-02; Syntech). Ground and recording electrodes consisted of a silver wire inserted into a pulled borosilicate glass capillary (i.d. 0.86 mm, Warner Instruments, Hamden, CT 06514) containing 0.1 M KCl as electrolyte with 1% polyvinylpyrrolidone. Wings and legs of the insect were removed, and the whole body placed into the ground electrode, leaving the antennae protruding. Contact was made between the recording electrode and the tips of both antennae, and the preparation was placed 5 mm from the GC outlet. GC and EAG signals were collected and analyzed with EZChrom software (Elite v3.0; Scientific Software, Pleasanton, CA, USA, now Agilent).

### Analysis by Gas Chromatography Coupled to Mass Spectrometry (GC–MS)

Collections were analyzed by GC–MS using a Varian 3700 GC linked directly to a Saturn 2200 ion-trap MS (Varian, now Agilent). Columns (30 m × 0.25 mm i.d. 0.25 μm film thickness) were coated with polar DBWax (Supelco) or non-polar VF5 (Varian/Agilent). Injection was splitless (220 °C), the carrier gas was helium (1 ml/min) and the oven temperature was held at 40 °C for 2 min then programmed at 10 °C/min to 250 °C and held for 5 min. Scan range was from *m/z* 40 to *m/z* 400. Retention Indices (RI) for compounds were calculated relative to the retention times of *n*-alkanes.

### Analysis by Enantioselective Gas Chromatography

Enantioselective GC analyses were carried out on a capillary column (25 m × 0.32 mm i.d. × 0.25 μm film thickness) coated with a cyclodextrin stationary phase (Chirasil-DEX CB; Varian, Oxford, UK). Injection was splitless (220 °C), detection was by FID (250 °C), and carrier gas was helium (2.4 ml/min). For analysis of volatile collections, the oven temperature was programmed from 50 °C for 2 min, then at 10 °C/min to 200 °C. For analysis of synthetic compounds, split injection was used and the oven temperature was held isothermally at 140 °C.

### Synthetic Chemicals

**Fig. 1:**
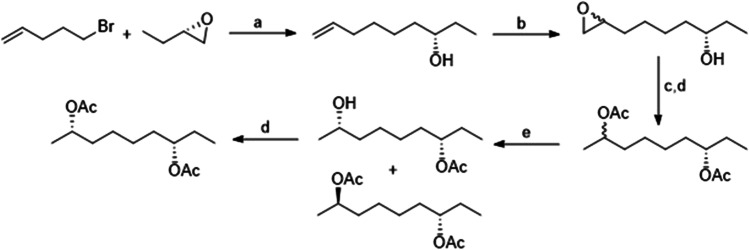
Synthesis of (2*R*,7*S*)-2,7-diacetoxynonane. Reagents (**a**) Mg, THF, I_2_, 1 h; CuI, THF, reflux, 3 h (65%); (**b**) m-CPBA, DCM (72%); (**c**) LiAlH_4_, Et_2_O; (**d**) Ac_2_O, Pyridine (67%); (**e**) *Candida antarctica* Lipase, 0.1 M K_2_HPO_4_ (aq) (85%)

The four diastereoisomers of 2,7-diacetoxynonane were synthesized as outlined for (2*R*,7*S*)-2,7-diacetoxynonane in Fig. 1 with full details in the Supplementary Material. The stereochemistry at C-7 was defined by use of the corresponding, commercially-available enantiomer of 1,2-epoxybutane, and that at C-2 by kinetic enzymatic resolution of the acetate using polymer supported lipase from *Candida antarctica* to give both enantiomers as reviewed in Hall et al. (2012). The enantiomeric excesses (e.e.) were determined by GC analysis on the cyclodextrin column and were as follows: (2*R,*7*S*)-isomer 97.3%, (2*S,*7*S*)- 96.4%, (*2R,7R*)- 98.4%, and (*2S,7R*)- 94.4%.

Racemic 2,8-diacetoxynonane was prepared by reaction of the di-Grignard reagent from 1,5-dibromopentane with acetaldehyde followed by acetylation of the resulting diol with acetic anhydride and pyridine, as described in the Supplementary Material.

The enantiomers of 2-nonanol were available from previous work (Rowley et al. 2017) by enzymatic kinetic resolution of commercially-available 2-nonanol. Acetylation gave (2*R*)- and (2*S*)-2-acetoxynonane with e.e. of 98.9% and 98.7%, respectively.

Racemic 3-acetoxynonane was prepared by acetylation of 3-nonanol with acetic anhydride and pyridine, as described in the Supplementary Material.

### Field Trapping Experiments

Field trials were conducted at 10 sites in northeastern Saskatchewan in July 2018 (Experiment 1) and June–August 2019 (Experiments 2 and 3). The fields were located near the towns of Melfort, Nipawin, and Arborfield (53.104, -103.661). Traps were deployed in a randomized complete block design (site = block) along the field edge, 25 m apart, and 0.5 m above the soil surface to trap midges emerging from the soil (Hall et al. 2012).

Traps were white Jackson traps (Distributions Solida, Saint-Ferréol-les-Neiges, Québec, Canada) and pheromone dispensers were closed polyethylene vials (26 mm × 8 mm × 1.5 mm thick; Just Plastics Ltd., London, UK). The latter were found to give more sustained release than rubber septa with compounds of similar molecular weight to those tested here (Rowley et al. 2017). The vials were impregnated by applying the pheromone in hexane solution containing 10% 2,6-di-*tert*-butyl-4-methylphenol (BHT) as antioxidant (100 µl), allowing the solvent to evaporate fully and then capping the vial. Lures were prepared in the UK, stored in heat-sealed aluminium foil bags, and shipped to Canada where they were kept in a refrigerator (4 °C) before use.

In Experiment 1, catches of *C. brassicola* were compared in traps baited with each of the four isomers of 2,7-diacetoxynonane individually (10 µg), the racemic mixture of the four isomers (40 µg) and an unbaited trap. After one week, traps, inserts, and lures were removed and replaced, and treatment position randomized within blocks. Numbers of male and female *C. brassicola* on each trap were counted each week, with the experiment repeated over two weeks from 28 June to 12 July 2018.

In Experiment 2, the effects of adding possible minor components (2*R*,7*R*)-diacetoxynonane and (2*R*)-2-acetoxynonane to the proposed major pheromone component, (2*R*,7*S*)-diacetoxynonane were investigated. Numbers of *C. brassicola* caught were compared between traps baited with dispensers loaded with one of five treatments: solvent only control; 10 µg (2*R,*7*S*)-2,7-diacetoxynonane; 10 µg (2*R,*7*S*)-2,7-diacetoxynonane plus 1 µg, 5 µg or 10 µg (2*R,*7*R*)-2,7-diacetoxynonane; and 10 µg (2*R,*7*S*)-2,7-diacetoxynonane plus 10 µg (2*R,*7*R*)-2,7-diacetoxynonane and 1 µg (2*R*)-2-acetoxynonane. The experiment ran from 27 June to 7 August 2019 and lures were changed once on 11 July. Traps were monitored and sticky liners replaced at ca. 1-week intervals, and treatment positions randomized. Numbers of male and female *C. brassicola* on each trap were counted each week.

In Experiment 3, the ratio of pheromone components was further refined, based on the treatments which caught the most male *C. brassicola* in Experiment 2. Traps were baited with dispensers loaded with one of seven treatments: solvent only control; 10 µg (2*R,*7*S*)-2,7-diacetoxynonane plus 0.1 µg, 0.5 µg, 1 µg, 2 µg or 5 µg (2*R,*7*R*)-2,7-diacetoxynonane; and 10 µg (2*R,*7*S*)-2,7-diacetoxynonane plus 1 µg (2*R,*7*R*)-2,7-diacetoxynonane and 0.5 µg (2*R*)-2-acetoxynonane. The experiment ran from 8–28 August 2019. Traps were monitored and sticky liners replaced at ca. 1-week intervals, and treatment positions randomized. Numbers of male and female *C. brassicola* on each trap were counted each week.

### Statistical Analyses

General linear mixed models were used to compare numbers of male *C. brassicola* caught in the different treatments (Bates et al. 2015). Numbers of males caught per trap per week were transformed to log(n + 1) and entered as the dependent variable and treatment entered as an independent factor (Experiment 1: six levels, Experiment 2: seven levels). Trap week and field site were entered as random factors. Significance of the treatment term within each model was assessed through χ^2^ tests of changes in residual deviance following deletion from the model (Pinheiro and Bates 2000). The significance of differences (*P* < 0.05) between catches with different lures in each experiment were assessed using Tukey’s post-hoc tests on estimated marginal means (Lenth 2020). All data analyses were performed in R 4.0.2 (R Core Team 2020).

## Results

### Pheromone Identification

**Fig. 2:**
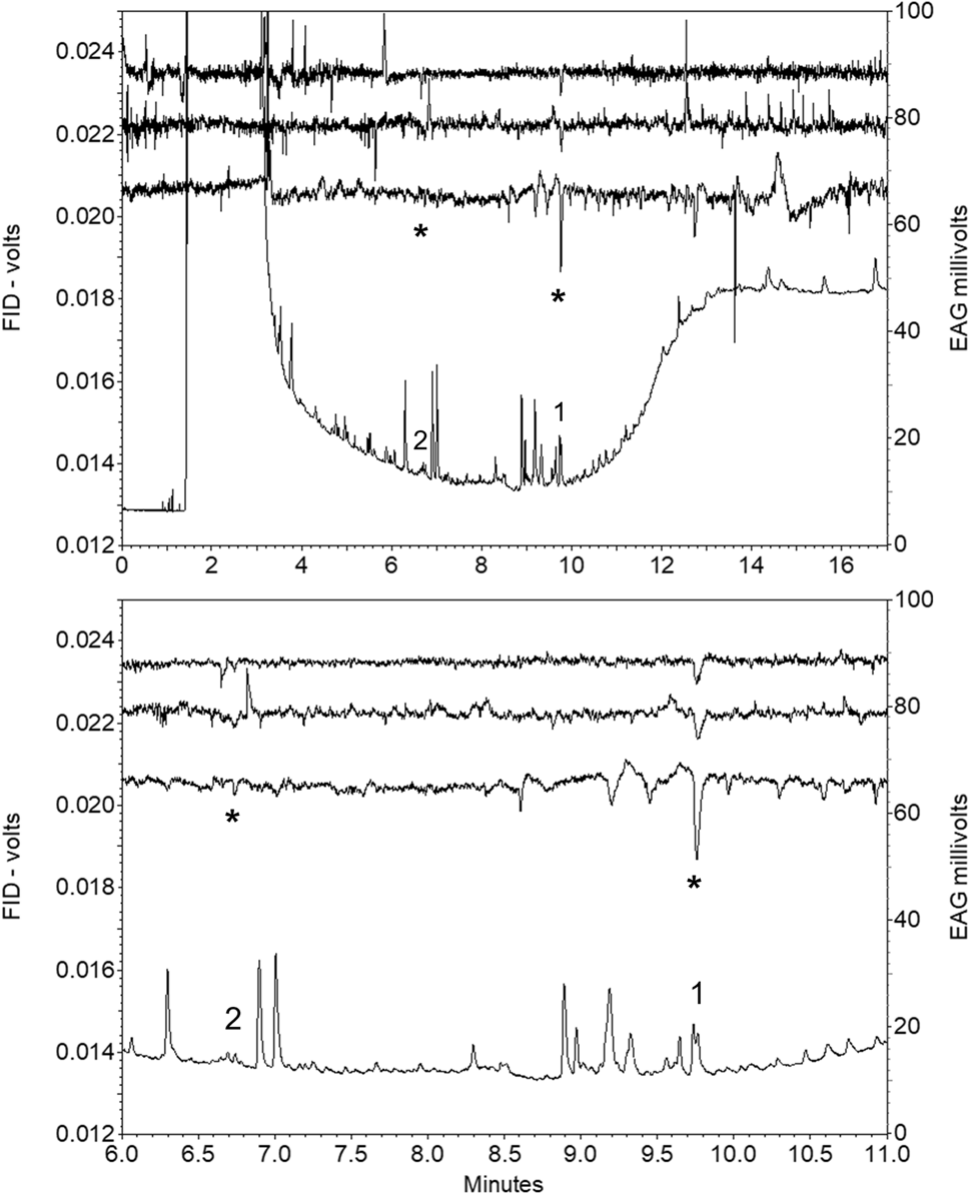
GC-EAG analyses of volatile collection from virgin female *Contarinia brassicola* with male *C. brassicola* EAG preparation on polar GC column showing EAG responses (*) to compounds proposed as major (1) and minor (2) pheromone components; lower chromatogram is expansion of upper; in each chromatogram lower trace is FID, upper traces EAG responses from three different males

In GC-EAG analyses of volatile collections from virgin female *C. brassicola* on a polar DBWax GC column with antennae of virgin male *C. brassicola,* two consistent EAG responses were observed (Fig. 2). These had retention indices (RI) of 1443 and 1968, with the latter response larger than the former, corresponding to an apparently larger peak in the FID chromatogram (Fig. 2). The compounds responsible for these responses were assumed to be minor and major components, respectively, of the female-produced sex pheromone of *C. brassicola.*

**Fig. 3:**
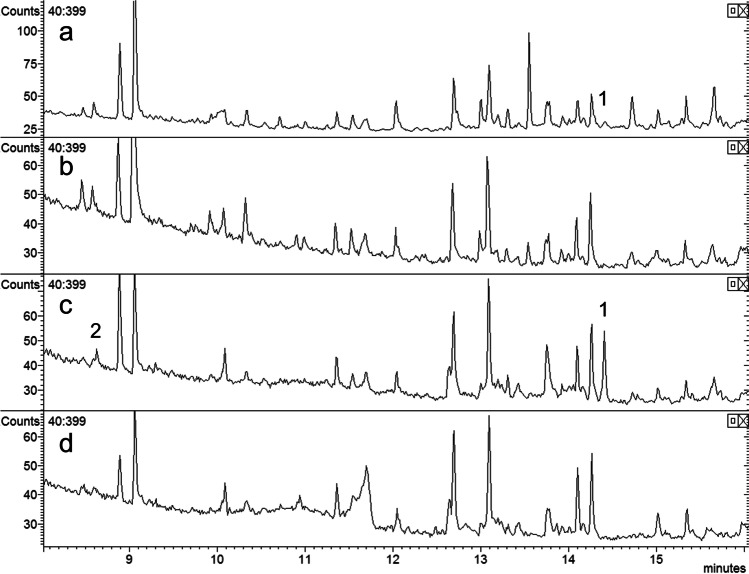
GC–MS analyses on polar GC column of collections of volatiles from (**a**) 2 females; (**b**) 1 male; (**c**) 68 females; (**d**) 34 males of *Contarinia brassicola.* Female-specific peaks proposed as major and minor components of the female-produced sex pheromone shown as 1 and 2, respectively

**Fig. 4:**
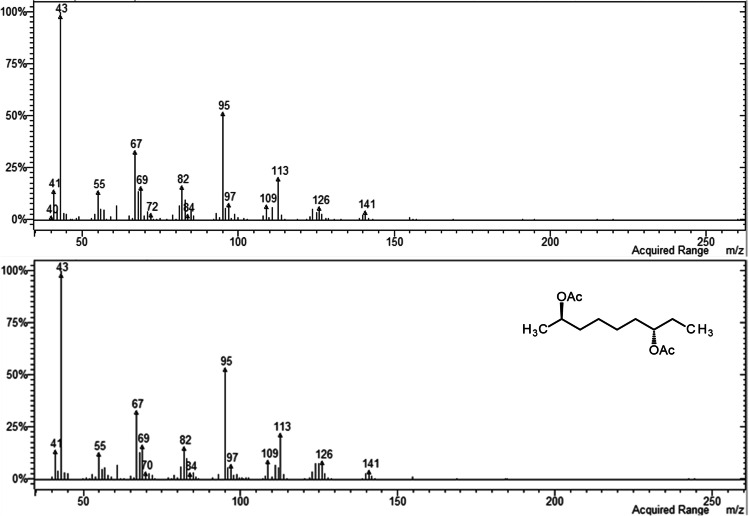
Mass spectrum of major female-specific compound in volatiles from *Contarinia brassicola* (upper) and synthetic (2*R*,7*S*)-2,7-diacetoxynonane (lower)

In analyses of the volatile collections by GC–MS on a similar polar DBWax GC column, a female-specific peak was observed at RI 1968 (Fig. 3) for the major pheromone component. This compound had the mass spectrum shown in Fig. 4, which was remarkably similar to those reported for 2,7-diacetoxyundecane, major component of the sex pheromone of the pear midge, *C. pyrivora* (Riley) (Amarawardana 2009; Hall et al. 2012), 2,7-dibutyroxynonane, sex pheromone of the orange wheat blossom midge, *Sitodiplosis mosellana* (Géhin) (Gries et al. 2000; Hooper et al. 2007), and 2,7-diacetoxytridecane, sex pheromone of the aphidophagous gall midge, *Aphidoletes aphidimyza* (Rondi) (Choi et al. 2004).

The RI 1968 indicated the compound had two fewer carbon atoms than 2,7-diacetoxyundecane (RI 2167; Amarawardana 2009), and the 2,7-diacetoxynonane structure was consistent with the mass spectrum (Fig. 4). Fragmentation ions at *m/z* 43 and 61 suggested the presence of acetate group(s). The ion at *m/z* 126 corresponded to the loss of two acetoxy groups from a just-detectable molecular ion at *m/z* 244, and that at *m/z* 124 to the loss of two acetic acid molecules from the molecular ion. Loss of an ethyl group from the latter would give the strong ion at *m/z* 95, providing evidence for one of the acetate groups at C-7. Loss of a methyl group from *m/z* 124 would give the ion at *m/z* 109 confirming the position of the other acetate group at C-2, in line with all midge pheromones reported to date which have an oxygen functionality at C-2 (Hall et al. 2012; Xu et al. 2020). Loss of one molecule of acetic acid from the molecular ion followed by loss of acetyl would give the ion at *m/z* 141, and subsequent loss of a terminal ethylene would account for the ion at *m/z* 113.

**Fig. 5:**
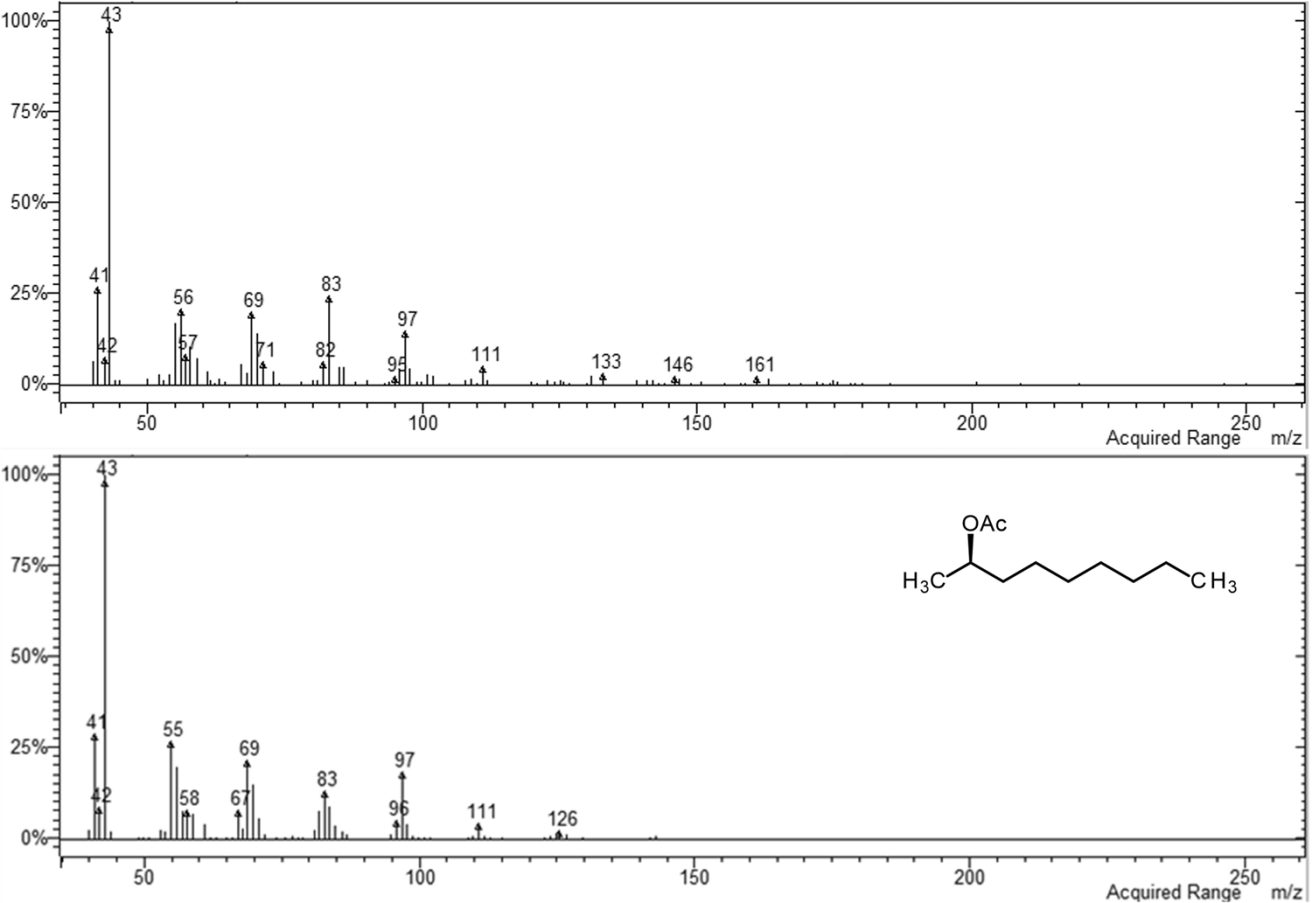
Mass spectrum of minor female-specific compound in volatiles from *Contarinia brassicola* (upper) and synthetic (2*R*)-2-acetoxynonane (lower)

The retention index and mass spectrum of the less abundant, minor pheromone component at RI 1443 (Fig. 5) were consistent with those expected for a 9-carbon monoacetate with ions at *m/z* 43 and 61 indicating an acetate group, and an ion at *m/z* 126 formed by loss of acetic acid from the molecular ion. The ion at *m/z* 111 formed by loss of methyl from *m/z* 126 suggested the acetate substitution was at C-2.

Both compounds could be detected in GC–MS analyses of collections of volatiles from virgin female *C. brassicola* on a non-polar VF5 column with RI 1533 and 1235 for the major and minor components, respectively.

Comparison of these data with those of synthetic standards showed that the mass spectrum and retention indices of the major pheromone component were identical with those of the later eluting diastereoisomer of synthetic 2,7-diacetoxynonane (RI on polar GC column 1965 and 1968). The mass spectrum and retention indices were different from those of synthetic 2,8-diacetoxynonane (mass spectrum Fig. S2 in Supplementary Material; RI 2007 and 1551 on polar and non-polar GC columns, respectively), and this latter compound could not be detected in volatiles from female *C. brassicola*. The mass spectrum and retention indices of the minor pheromone component were identical with those of 2-acetoxynonane and different from those of 3-acetoxynonane (mass spectrum Fig. S3 in Supplementary Material; RI 1416 and 1219 on polar and non-polar GC columns, respectively), the other monoacetoxynonane corresponding to 2,7-diacetoxynonane. In particular, the mass spectrum of 3-acetoxynonane lacked the ion at *m/z* 111 apparent in that of the minor pheromone component, and showed a very strong ion at *m/z* 97 formed by loss of acetic acid and an ethyl group. 3-Acetoxynonane could not be detected in volatiles collected from female *C. brassicola.* Retention indices of natural and synthetic compounds are summarized in Table S1 of the Supplementary Material.

**Fig. 6:**
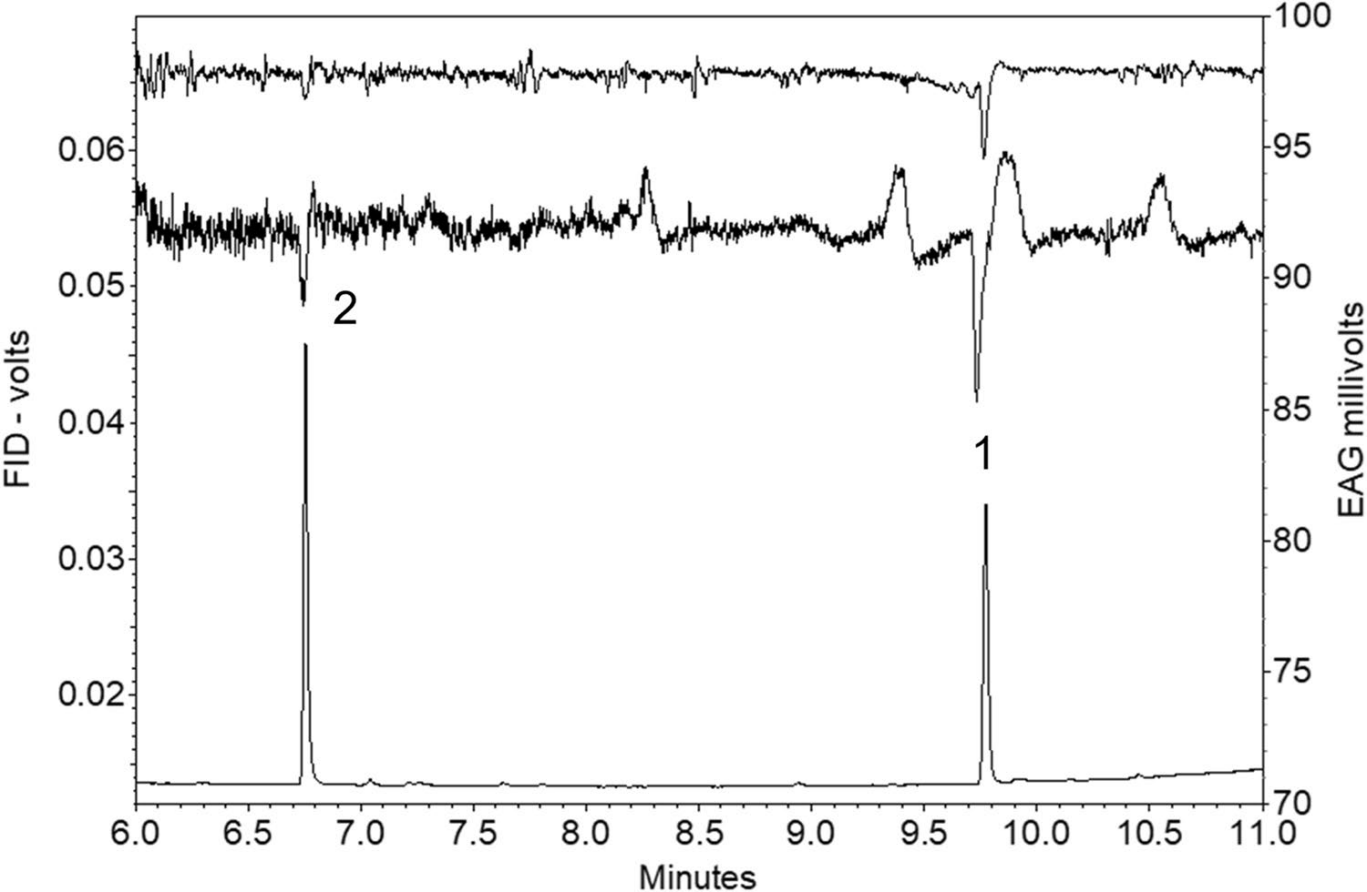
GC-EAG analyses with male *Contarinia brassicola* EAG preparation on polar GC column showing EAG responses to synthetic 2,7-diacetoxynonane (1) and 2-acetoxynonane (2) (approx 10 ng injected); lower trace is FID, upper traces EAG responses from two different males

Synthetic racemic 2,7-diacetoxynonane and 2-acetoxynonane elicited strong EAG responses from antennae of male *C. brassicola* (Fig. 6).

### Pheromone Synthesis and Stereochemistry

**Fig. 7:**
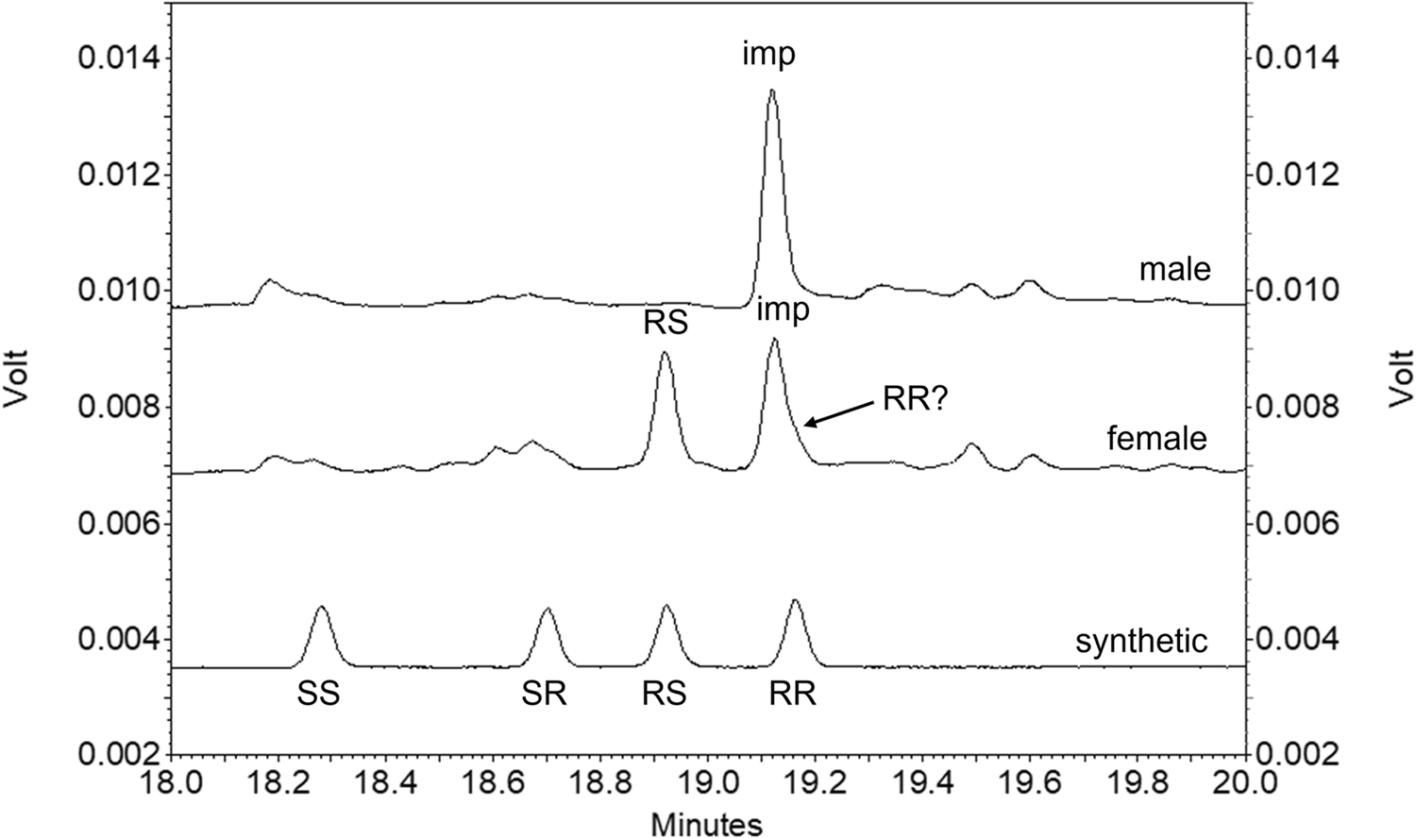
GC-FID Analyses on enantioselective cyclodextrin column of (from bottom) synthetic isomers of 2,7-diacetoxynonane, collection of volatiles from 68 female *Contarinia brassicola* and collection of volatiles from 34 male *C. brassicola;* “imp” indicates impurity in collections from both males and females; in analysis of collection from females, “RS” indicates major female-specific component and “RR?” indicates possible minor female-specific component

The four stereoisomers of 2,7-diacetoxynonane were synthesized and clearly separated in GC analyses on an enantioselective cyclodextrin column (Fig. 7). The order of elution confirmed that reported by Hooper et al. (2007), with the separation of the C-2 enantiomers being greater than that for the C-7 enantiomers. Analyses on a polar GC column showed that the first eluting peak contained the *threo*- ((2*R,*7*R*)- and (2*S,*7*S*)-) and the second peak the *erythro*-diastereoisomers ((2*R,*7*S*)- and (2*S,*7*R*)-).

Analysis of collections of volatiles from *C. brassicola* on the cyclodextrin GC column showed a female-specific peak at the retention time of (2*R,*7*S*)-2,7-diacetoxynonane (Fig. 7), consistent with results of analyses on the polar GC column in which the female specific peak corresponded to the second peak containing the *erythro*-diastereoisomers. No peaks were consistently detected corresponding to the (2*S,*7*S*)- or (2*S,*7*R*)-isomers, but an impurity peak was present in collections from both males and females at the retention time of the (2*R,*7*R*)-isomer (Fig. 7).

The enantiomers of the minor pheromone component, 2-acetoxynonane, were also well-separated on the cyclodextrin column (2*S*- and 2*R*- 11.60 min and 12.60 min, respectively), but the configuration of this compound in volatile collections from female *C. brassicola* could not be reliably determined because of the presence of impurity peaks in similar, small amounts. It was anticipated to be the (2*R*)-enantiomer, bearing in mind the stereochemistry of the major component.

**Fig. 8:**
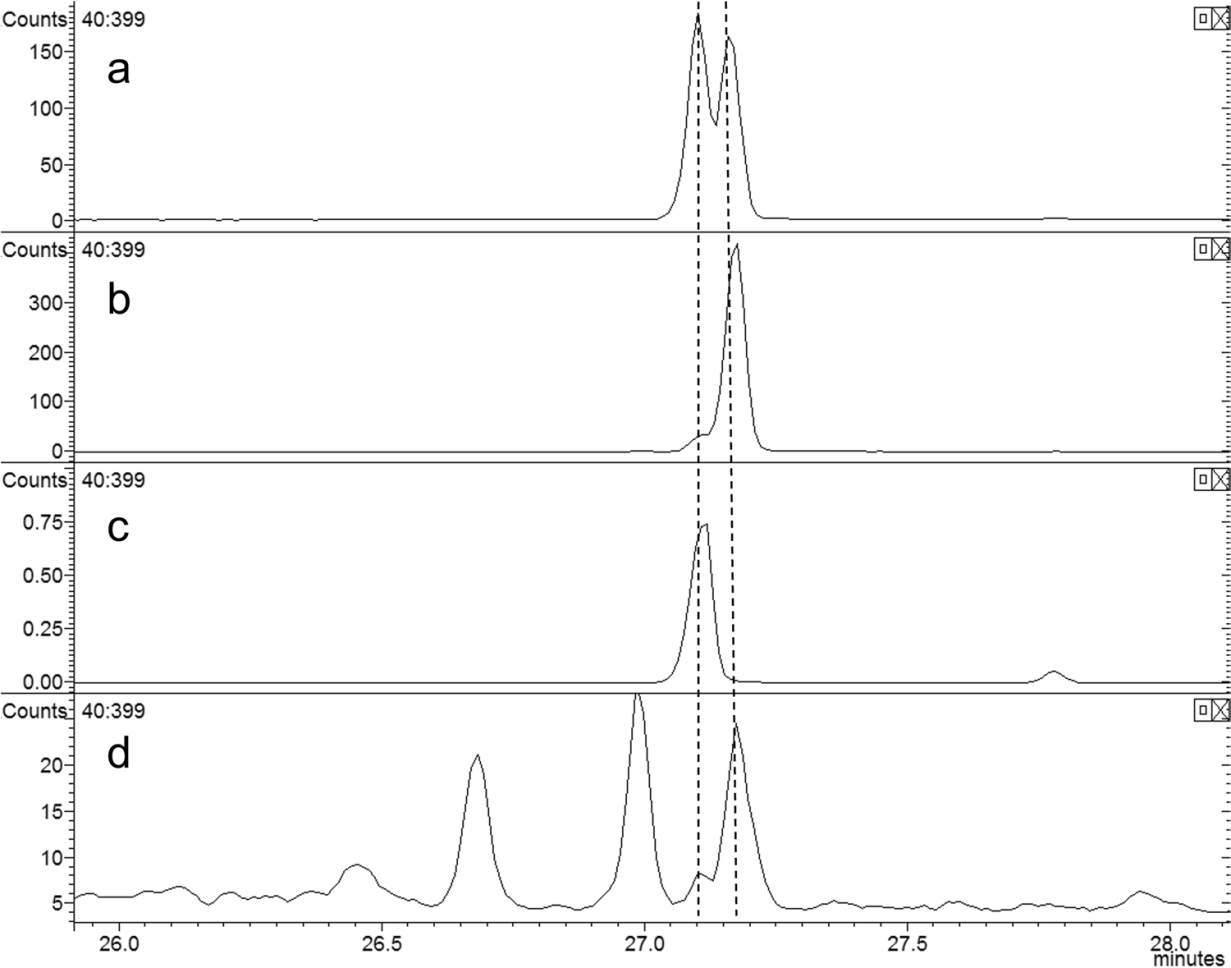
GC–MS Analyses on polar GC column with slow temperature program (5 °C/min) of (**a**) racemic 2,7-diacetoxyonane, (**b**) (2*R*,7*S*)-2,7-diacetoxynonane, (**c**) (2*R*,7*R*)-2,7-diacetoxynonane, and (**d**) collection of volatiles from *Contarinia brassicola* showing presence of both diastereoisomers of 2,7-diacetoxynonane

In the first field test described below, traps baited with each of the individual stereoisomers or the racemic 2,7-diacetoxynonane caught very few male *C. brassicola*. This prompted a re-examination of the analytical data and it was realized that in analyses on the cyclodextrin GC column, the impurity peak in volatiles from both male and female *C. brassicola* eluted slightly earlier than (2*R,*7*R*)-2,7-diacetoxynonane, and that a later-eluting shoulder on this impurity peak at the retention time of the latter isomer could be detected in volatile collections from the female midges that was not present in volatile collections from males (Fig. 7). Similarly, in GC–MS analyses of collections of volatiles on the polar GC column using a slower temperature program (5 °C/min rather than 10 °C/min) (Fig. 8), a peak corresponding to the earlier eluting, *threo*-diastereoisomers of 2,7-diacetoxynonane could be detected with the appropriate mass spectrum.

It was thus concluded that likely components of the sex pheromone produced by female *C. brassicola* are (2*R,*7*S*)-2,7-diacetoxynonane, (2*R,*7*R*)-2,7-diacetoxynonane and (2*R*)-2-acetoxynonane in 100: 10: 5 ratio. The best collection of volatiles from 68 virgin female *C. brassicola* over 72 h contained approximately 25 ng (2*R,*7*S*)-2,7-diacetoxynonane.

### Field Trapping Experiments

In Experiment 1, only a very small number of male *C. brassicola* were caught in traps baited with dispensers loaded with individual isomers of 2,7-diacetoxynonane over two weeks ((2*R,*7*R*)- total two males; (2*R,*7*S*)- eight males; (2*S,*7*R*)- zero males; (2*S,*7*S*)- two males). Traps baited with the racemic mixture caught four male *C. brassicola*, and unbaited traps caught zero males. These numbers were too low for formal analysis. Traps also caught female *C. brassicola* ((2*R,*7*R*)- total 23 females; (2*R,*7*S*)- 31 females; (2*S,*7*R*)- 57 females; (2*S,*7*S*)- 33 females; racemic mixture- 42 females; unbaited traps- 47 females) with an overall mean of 1.9 females/trap/week. There was no significant effect of lure treatment on numbers of female *C. brassicola* caught (Mixed Model, χ^2^ = 3.97, df = 5, *P* = 0.55).

**Fig. 9:**
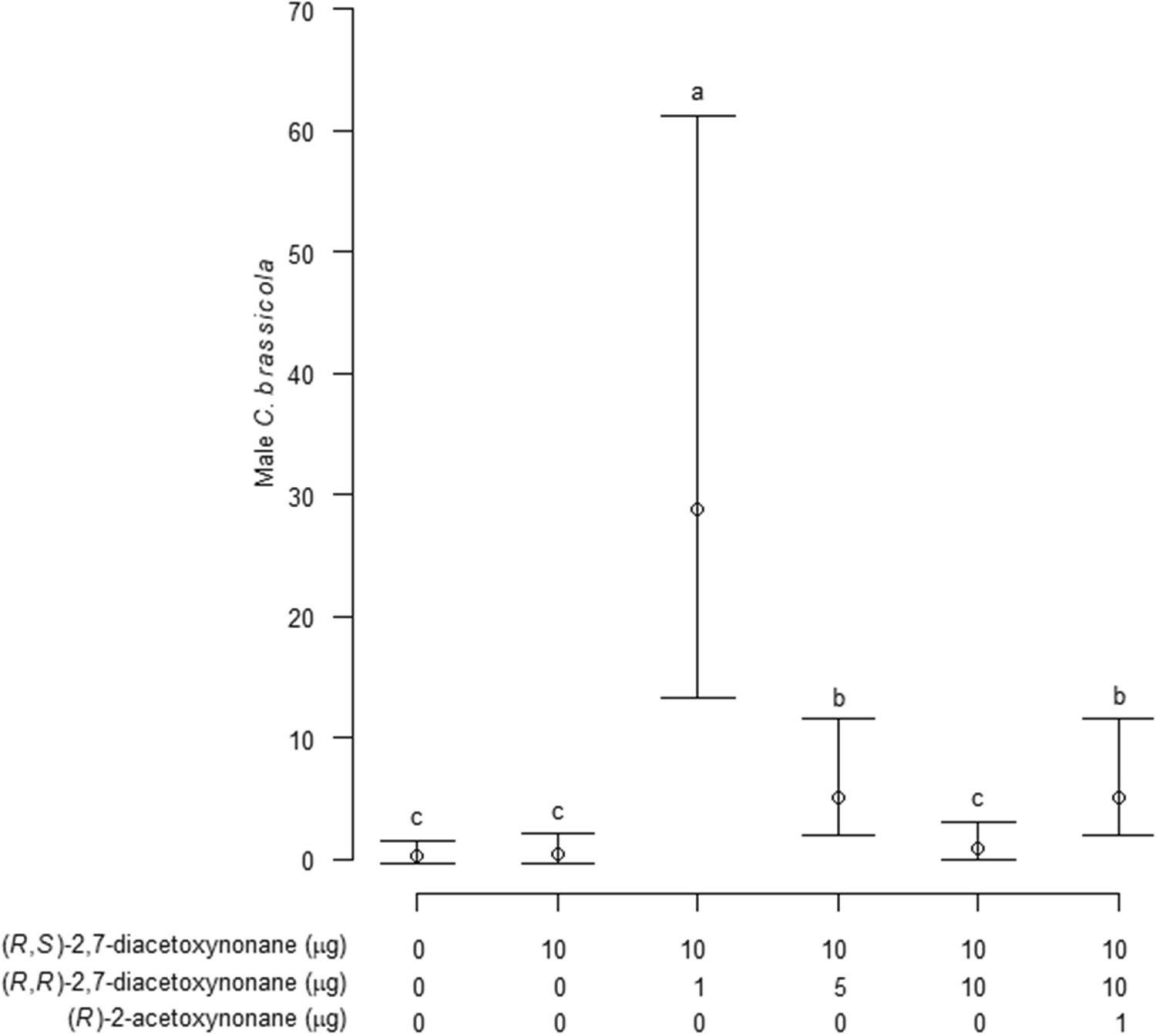
Mean number (± 95% confidence interval) of male *Contarinia brassicola* caught per trap per week with dispensers loaded with five different ratios of two isomers of 2,7-diacetoxynonane and (2*R*)-2-acetoxynane plus an unbaited control in Experiment 2. Means are estimated marginal means based on the fixed effects of the mixed model used for analysis, with means and confidence intervals back-transformed from the logarithmic scale. Different letters indicate significant differences in numbers of midges caught (*P* < 0.05)

In Experiment 2, a significant overall difference was found in numbers of males caught in traps baited with the different lure treatments (Mixed Model, χ^2^ = 253, df = 5, *P* < 0.001; Fig. 9). Traps baited with lures loaded with 10 µg (2*R,*7*S*)-2,7-diacetoxynonane plus 1.0 µg (2*R,*7*R*)-2,7-diacetoxynonane caught the most male *C. brassicola*, and significantly more males than those baited with lures loaded with 10 µg (2*R,*7*S*)-2,7-diacetoxynonane plus 5 µg (2*R,*7*R*)-2,7-diacetoxynonane or 10 µg (2*R,*7*S*)-2,7-diacetoxynonane plus 10 µg (2*R,*7*R*)-2,7-diacetoxynonane plus 1 µg (2*R*)-2-acetoxynonane (Tukey’s test. *P* < 0.05). Traps baited with the two latter treatments caught significantly more males than those baited with 10 µg (2*R,*7*S*)-2,7-diacetoxynonane alone, 10 µg (2*R,*7*S*)-2,7-diacetoxynonane and 10 µg (2*R,*7*R*)-2,7-diacetoxynonane, or the solvent only control. A small number of female *C. brassicola* were caught (mean 0.9 females/trap/week; maximum 11 females on a single trap) and there were no significant differences in the numbers of females caught between treatments (Mixed Model, χ^2^ = 7.44, df = 5, *P* = 0.19).

**Fig. 10:**
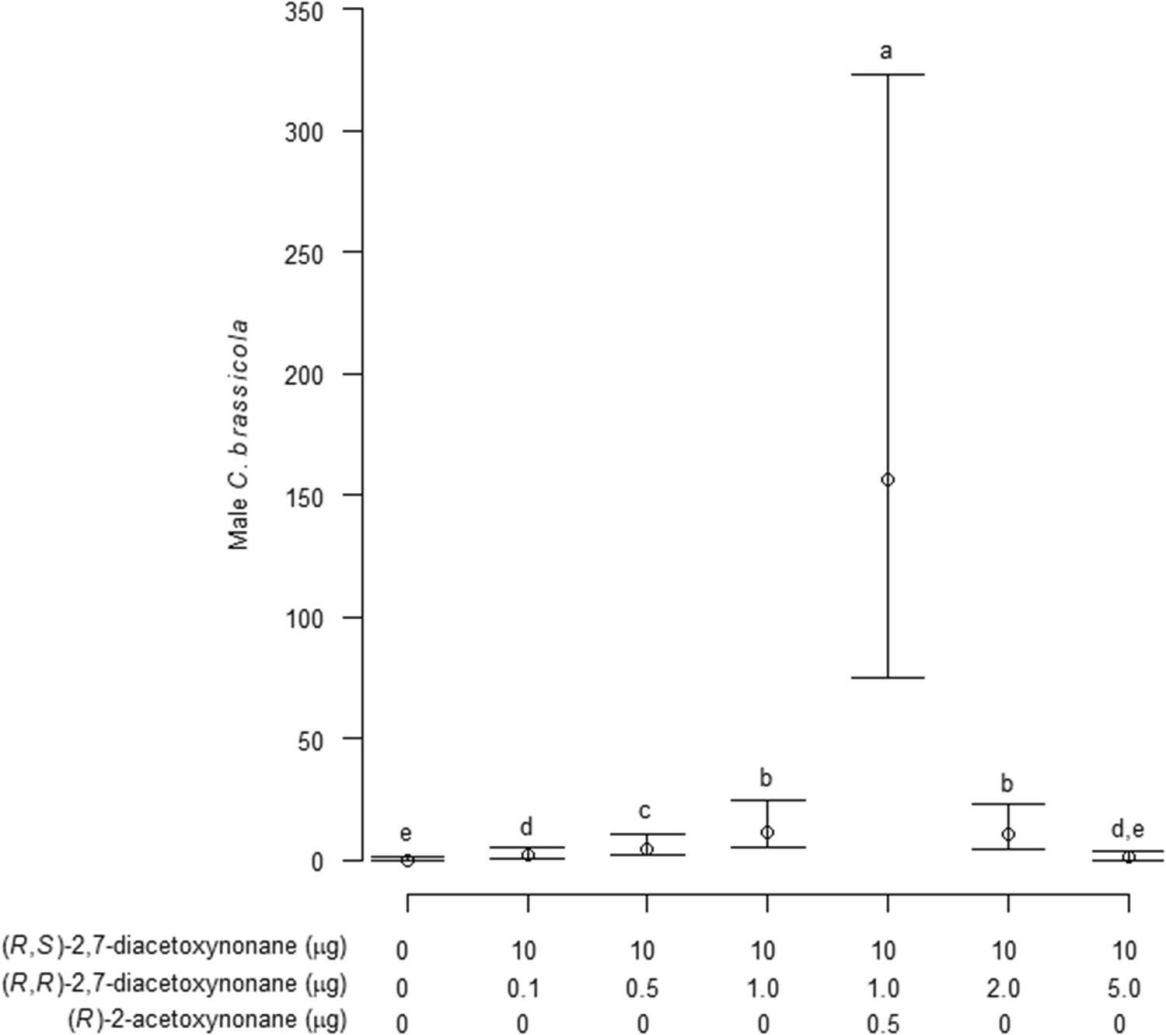
Mean number (± 95% confidence interval) of male *Contarinia brassicola* caught per trap per week with dispensers loaded with six different ratios of (2*R*,7*S*)- and (2*R*,7*R*)-2,7-diacetoxynonane and (2*R*)-2-acetoxynane and an unbaited control in Experiment 3. Means are estimated marginal means based on the fixed effects of the mixed model used for analysis, with means and confidence intervals back-transformed from the logarithmic scale. Different letters indicate significant differences in numbers of midges caught (*P* < 0.05)

In Experiment 3, a significant overall difference was found between treatments in numbers of males caught (Mixed Model, χ^2^ = 308, df = 6, *P* < 0.001; Fig. 10), with differences between individual treatments detected through Tukey’s tests (*P* < 0.05). Traps baited with 10 µg (2*R,*7*S*) -2,7-diacetoxynonane plus 1.0 µg (2*R,*7*R*)-2,7-diacetoxynonane and 0.5 µg (2*R*)-2-acetoxynonane caught the most males and significantly more males than traps baited with 10 µg (2*R,*7*S*) -2,7-diacetoxynonane and 1.0 µg (2*R,*7*R*)-2,7-diacetoxynonane or 10 µg (2*R,*7*S*)-2,7-diacetoxynonane and 2.0 µg (2*R,*7*R*)-2,7-diacetoxynonane. Traps baited with the two latter treatments caught more males than traps baited with 10 µg (2*R,*7*S*)-2,7-diacetoxynonane and 0.5 µg (2*R,*7*R*) -2,7-diacetoxynonane, which in turn caught males than traps baited with 10 µg (2*R,*7*S*)-2,7-diacetoxynonane and 0.1 µg (2*R,*7*R*)-2,7-diacetoxynonane or 10 µg (2*R,*7*S*)-2,7-diacetoxynonane and 5 µg (2*R,*7*R*)-2,7-diacetoxynonane. There was no difference in the numbers of males caught in traps baited with lures loaded with 10 µg (2*R,*7*S*)-2,7-diacetoxynonane and 5 µg (2*R,*7*R*)-2,7-diacetoxynonane and the blank control. Few female *C. brassicola* were caught (mean 0.73 females/trap/week, maximum 10 females on a single trap), and there was no significant overall difference in the number of females caught between treatments (Mixed Model, χ^2^ = 4.8, df = 6, *P* = 0.57).

## Discussion

The results of this study demonstrated that virgin female *C. brassicola* produced a sex pheromone consisting of three components: (2*R,*7*S*)-2,7-diacetoxynonane, (2*R,*7*R*)-2,7-diacetoxynonane and (2*R*)-2-acetoxynonane. In field trapping tests, the individual components did not attract male *C. brassicola*, and the most attractive blend of those tested was similar to that produced by the female midges in a 100: 10: 5 ratio, respectively.

The major component of the pheromone of *C. brassicola,* 2,7-diacetoxynonane, has not been reported as a component of the sex pheromone of any other cecidomyiid midge, but has a structure consistent with those found in many other midge species (Hall et al. 2012; Xu et al. 2020). The corresponding dibutyrate is the sex pheromone of the orange blossom wheat midge, *S. mosellana* (Gries et al. 2000). The 2,7-diacetoxy motif is found in 2,7-diacetoxyundecane, a component of the sex pheromone of the pear midge, *C. pyrivora* (Amarawardana 2009; Hall et al. 2012), and 2,7-diacetoxytridecane, sex pheromone of the aphidophagous gall midge, *A. aphidimyza* (Choi et al. 2004). Among other *Contarinia* species, the sex pheromone of the pea midge, *C. pisi* includes 2,11-diacetoxytridecane (Hillbur et al. 1999, 2000, 2001), and that of the swede midge, *C. nasturtii* includes 2,9-diacetoxyundecane (Boddum et al. 2009; Hillbur et al. 2005). These are structurally related to the 2,7-diacetoxynonane in the *C. brassicola* pheromone in having acetoxy groups at C-2 and at the third carbon in from the other end of the molecule. The pheromones of *C. pisi* and *C. nasturtii* also include symmetrical 2,12-diacetoxytridecane (Hillbur et al. 1999, 2000, 2001) and 2,10-diacetoxyundecane (Boddum et al. 2009; Hillbur et al. 2005), respectively. The analogous 2,8-diacetoxynonane could not be detected in volatiles from female *C. brassicola.* Thus, whereas the sex pheromones of *C. pisi* and *C. nasturtii* each include two diacetate positional isomers, that of *C. brassicola* relies on two diacetate stereoisomers. This may reflect the closer phylogenetic relationship of *C. pisi* and *C. nasturtii* which appear to be sister species (Molnar et al. 2018), with *C. brassicola* sister to the clade containing both *C. pisi* and *C. nasturtii* (Mori et al. 2019). The sex pheromone of the only other *Contarinia* species for which a pheromone has been identified, the douglas-fir cone gall midge, *C. oregonensis* (Foote), is rather different in structure, being reported as (4*Z,*7*Z*)-2-acetoxy-4,7-tridecadiene by Gries et al. (2002).

2-Acetoxynonane has been reported to be a component of the alarm pheromone of the honey bee, *Apis mellifera* L. (Collins and Blum 1983) and of the venom of several paper wasp species, *Polistes* spp. (Bruschini et al. 2006). It has not previously been found as a component of the sex pheromone of a cecidomyiid midge, although (2*R*)-2-butyryloxynonane is the pheromone of the saddle gall midge, *Haplodiplosis marginata* (von Roser) (Censier et al. 2014; Rowley et al. 2017, 2018). In *C. brassicola*, addition of 5% (*R*)-2-acetoxynonane to the blend of (2*R*,7*S*)- and (2*R*,7*R*)-2,7-diacetoxynonane increased catches of male midges ten-fold. A similar situation was reported in *C. pisi* where addition of a small amount of (2*S*)-2-acetoxytridecane to the blend of (2*S,*11*S*)-2,11-diacetoxytridecane and (2*S,*12*S*)-2,12-diacetoxytridecane greatly increased catches of male midges (Hillbur et al. 1999, 2000, 2001).

Both stereochemistry and blend ratios are critical in the pheromone of *C. brassicola.* (2*R*,7*S*)-Diacetoxynonane is unattractive to male *C. brassicola*, as are the other individual stereoisomers. Addition of the (2*R*,7*R*)-isomer at 10–20% of the major component gives a highly attractive blend, but blends with lower or higher proportions are less attractive. This contrasts with the closely related 2,7-dibutyryloxynonane, sex pheromone of *S. mosellana,* where the (2*S*,7*S*)-isomer is produced by the female midges and the synthetic compound is attractive to male midges, but the racemic compound is equally attractive (Bruce et al. 2007; Gries et al. 2000). In other *Contarinia* species, only one stereoisomer is attractive and the racemic mixtures are not attractive, indicating one or more of the other stereoisomers inhibits attraction. In *C. pisi*, a blend of (2*S*,11*S*)- and (2*S*,12*S*)-diacetoxytridecane is attractive (Hillbur et al. 1999, 2000, 2001), and in *C. nasturtii* a blend of (2*S*,9*S*)- and (2*S*,10*S*)-diacetoxyundecane is attractive (Boddum et al. 2009; Hillbur et al. 2005). For *C. pyrivora*, (2*R*,7*R*)-diacetoxyundecane is attractive to male midges (Amarawardana 2009), but composition of the most attractive blend is currently under re-investigation (unpublished).

Some female *C. brassicola* were caught in all three trapping experiments, but there were no significant differences between the catches in traps baited with the different pheromone blends or the unbaited traps in any of the experiments. Similar numbers of females were caught in the three experiments, and catches of females were greater than catches of males in unbaited traps, possibly because of the stronger flight activity of the females in searching for oviposition sites.

Identification of the sex pheromone of *C. brassicola* will allow for future studies to determine the potential impact of this species on canola production in North America. Canola is a significant crop in Canada, and the damage caused by *C. brassicola* is discrete and difficult to recognize to the untrained eye. The availability of this pheromone will allow for monitoring of population dynamics and help to determine the pest status of this insect. Currently, it is hypothesized that *C. brassicola* is a native species to North America (Campbell et al. 2020; Mori et al. 2019). However, the availability of a pheromone trap will allow for future studies to determine if *C. brassicola* is found elsewhere throughout the world and may aid in the identification of other host plants. Studies have been commenced to determine the optimum trap design and placement for *C. brassicola*, and to attempt to correlate numbers of males captured in pheromone-baited traps with damage to canola in the field.

## Supplementary Information

Below is the link to the electronic supplementary material.

Supplementary file1 (PDF 333 KB)

## Data Availability

Raw chromatographic and trapping data are available on request from the authors.

## Code Availability

R Code used for data analysis is available from authors on request.
