# Supplementary material for: Components of the Female Sex Pheromone of the Newly-Described Canola Flower Midge, Contarinia brassicola
Source: J Chem Ecol. 2022 Jun 30;48(5-6):479–90. doi: 10.1007/s10886-022-01369-z (PMC9375766; doi:10.1007/s10886-022-01369-z)
Supplement: Supplementary file 1 — Supplementary file1 (PDF 333 KB) [file 10886_2022_1369_MOESM1_ESM.pdf]

Supplementary Material

Components of the Female Sex Pheromone of the Newly-described Canola Flower Midge, *Contarinia brassicola*

Daniel P Bray[1] David R Hall[1] Steven J Harte[1] Dudley I Farman[1] Meghan A Vankosky[2] Boyd A Mori[3]

[1] Natural Resources Institute, University of Greenwich, Chatham Maritime, Kent, UK

[2] Agriculture and Agri-Food Canada, Saskatoon, Canada

[3] Department of Agricultural, Food and Nutritional Science, University of Alberta, Edmonton, Canada

Daniel Bray: d.bray@gre.ac.uk

Additional Methods and Materials

Unless otherwise stated, all chemicals were obtained from SigmaAldrich (now Merck), Gillingham, Dorset, UK, and reactions were performed under a positive pressure of dry nitrogen. Analyses by GC-FID were carried out on a HP6850 GC (Agilent) fitted with a fused silica capillary column (30 m x 0.32 mm i.d. x 0.25 µm film thickness) coated with polar DBWax (Supelco). The oven temperature was held at 50 °C for 2 min then programmed at 10 °C/min to 250 °C and held for 5 min. Carrier gas was helium (2.4 ml/min), injection was splitless (220 °C) and detection by FID (250 °C). Retention indices were measured relative to the retention times of *n*-alkanes in GC-MS analyses as reported in the main text and summarized in Table S1 below.

Synthesis of Stereoisomers of 2,7-Diacetoxynonane (Fig. S1)

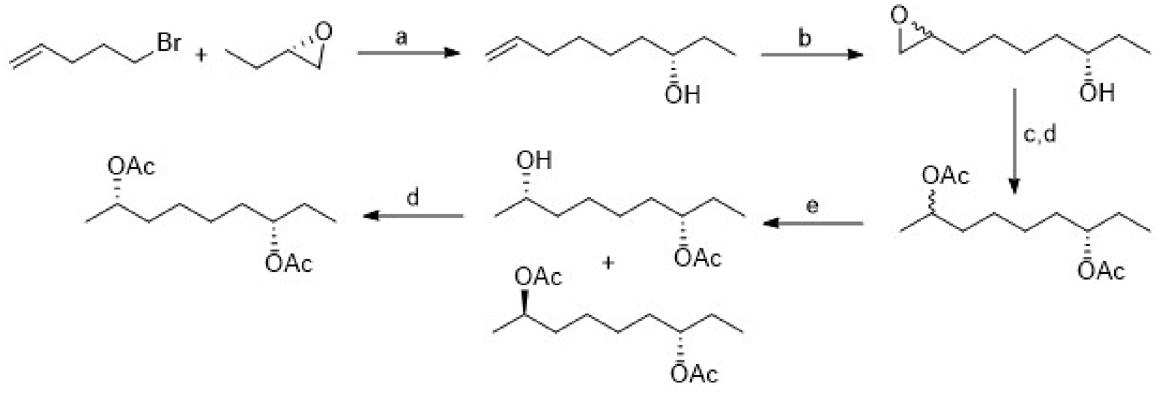

Fig. S1 Synthesis of (2*R*,7*S*)-2,7-diacetoxynonane. Reagents (a) Mg, THF, I_2, 1h; CuI, THF, reflux, 3 h (65%); (b): m-CPBA, DCM (72%); (c) $LiAlH_4$, Et_2O; (d) Ac_2O, Pyridine (67%); (e): *Candida antarctica* Lipase, 0.1M K_2HPO_4 (aq) (85%)

(7*S*)-1-Nonen-7-ol

Magnesium turnings (0.7 g, 28.8 mmol) were purged three times with vacuum followed by nitrogen, and anhydrous THF (10 ml) and a crystal of iodine were added. 5-Bromo-1-pentene (2 g, 13.4 mmol) was dissolved in anhydrous THF (5 ml) and added dropwise over 30 min with heating as appropriate. The mixture was then stirred for a further 2 h. CuI (133 mg, 0.70 mmol) was then added before (2*S*)-1,2-epoxy butane (0.5 g, 6.93 mmol) in anhydrous THF (5 ml), was added dropwise at -20 °Cu over 30 min. The mixture was then allowed to warm to room temperature and stirred until the reaction was complete by GC-FID analysis (approximately 3 h). The reaction was quenched with saturated aqueous NH_4Cl (10 ml), extracted with Et_2O (3 x 20 ml) and the organic phase was dried with anhydrous $MgSO_4$ and solvents were removed on a rotary evaporator. The product was isolated by column chromatography on silica gel (Et_2O, pet Ether) giving (7*S*)-1-nonen-7-ol (0.64 g, 4.50 mmol).

(7*S*)-1,2-Epoxy-7-nonanol

(7*S*)-1-Nonen-7-ol (0.5 g, 3.52 mmol) was dissolved in dry dichloromethane (10 ml) and cooled to 5 °C in an ice bath. m-CPBA (1.1 g, 7 mmol) was then added before the solution was allowed to warm to room temperature. The reaction was stirred for a further 2 h before being quenched

with aqueous KOH (1 M, 10 ml), extracted with Et_2O (3 x 20 ml) and the organic phase was dried with anhydrous $MgSO_4$ and solvents were removed on a rotary evaporator, resulting in (7*S*)-1,2-epoxy-7-nonanol (0.40 g, 2.53 mmol).

(7*S*)-2,7-Diacetoxynonane

(7*S*)-1,2-Epoxy-7-nonanol (0.25 g, 1.58 mmol) was dissolved in anhydrous Et_2O (5 ml) and cooled to 0 °C before $LiAlH_4$ (200 mg, 5.30 mmol) was added slowly. The reaction was stirred for 30 min at 0 °C before the mixture was allowed to warm to room temperature and stirred for a further 1 h. The reaction was quenched by the consecutive additions of H_2O (160 µl), KOH (1 M, 160 µl) and H_2O (3 x 160 µl). The mixture was filtered and extracted with Et_2O (3 x 5 ml), subsequently the organic phases were combined, dried with anhydrous $MgSO_4$ and evaporated. The crude product was dissolved in a 1:1 acetic anhydride:pyridine solution (2 ml). This mixture was stirred for 18 h before being quenched by aqueous $CuSO_4$ (0.1 M, 10 ml) and extracted with Et_2O (3 x 20 ml). The organic phases were then combined, dried with anhydrous $MgSO_4$ and solvents were removed on a rotary evaporator. (7*S*)-2,7-Diacetoxynonane (0.28 g, 1.14 mmol) was isolated by column chromatography on silica gel (Et_2O, pet Ether).

Enzymatic Kinetic Resolution of (7*S*)-2,7-Diacetoxynonane

(7*S*)-2,7-Diacetoxynonane (0.2 g, 0.82 mmol) was dissolved in aqueous K_2HPO_4 buffer (0.1 M, 2 ml) before immobilized lipase from *Candida antarctica* (20 mg) was added. The mixture was stirred for 2 h and the selective hydrolysis of the acetate was followed by GC-FID. Once 50% completion had been reached, the mixture was filtered and extracted with Et_2O (3 x 5 ml), and the organic phases were combined, dried with $MgSO_4$ and evaporated. The (2*R,*7*S*)-diacetoxynonane (83.1 mg, 0.34 mmol) and (2*S,*7*S*)-2-acetoxy-7-nonanol (72.7 mg, 0.36 mmol) were isolated by column chromatography on silica gel (Et_2O, pet Ether). (2*S,*7*S*)-2-Acetoxy-7-nonanol was re-acetylated in a 1:1 acetic anhydride:pyridine solution using the method detailed above to produce *(*2*S,*7*S)*-2,7-diacetoxynonane. The (2*R,*7*S*)- and (2*S,*7*S*)-2,7-diacetoxynonanes had enantiomeric excess (ee) of 97.3% and 96.4%, respectively, by GC analysis on the Chirasil-DEX CB column.

^1H NMR ($CDCl_3$, 400 MHz) δ 4.88 (q of t, *J* = 6.3, 7.2 Hz; 1H), 4.80 (t of t, *J* = 6.8, 5.2 Hz; 1H), 2.05 (s; 3H), 2.03 (s; 3H), 1.5-1.6 (m; 6H), 1.25-1.35 (m; 4H), 1.20 (d, *J* = 6.3 Hz; 3H), 0.88 (t,

J= 7.5 Hz, 3H). ^{13}C NMR ($CDCl_3$, 100 MHz) δ 170.99, 170.80, 75.34, 70.87, 35.81, 33.49, 26.95, 25.32, 25.17, 21.40, 21.26, 19.97, 9.59.

(2*R*,7*R*)-Diacetoxynonane (132.6 mg, 0.57 mmol) and (2*S*,7*R*)-diacetoxynonane (122.6 mg, 0.61 mmol) were obtained *via* the same methods using (2*R*)-1,2-epoxy butane and resulting in similar yields and an ee of 98.4% and 94.4%, respectively.

Synthesis of 2,8-Diacetoxynonane

Magnesium turnings (0.72 g, 30 mM) were washed with diethyl ether and dried under vacuum. Anhydrous THF (20 ml) was added under dry nitrogen along with a crystal of iodine. A few drops of a solution of 1,5-dibromopentane (2.3 g, 10 mM) in anhydrous THF (10 ml) were added to the stirred suspension until the reaction started. The remaining solution was added dropwise to maintain gentle reflux, and then stirred for 30 min at room temperature. The solution was stirred and cooled in ice and a solution of acetaldehyde (1.1 g, 25 mM) in anhydrous THF (5 ml) was added dropwise over 15 min. After bringing to room temperature over 1 h, the reaction was quenched with saturated aqueous NH_4Cl and the organic layer was separated and most of the THF was removed on a rotary evaporator. The residue was dissolved in diethyl ether/petroluem spirit (1:1), washed twice with saturated aqueous NH_4Cl and dried over anhydrous $MgSO_4$.

Removal of solvent gave 2,8-nonanediol (2.0 g) which was acetylated with acetic anhydride (3 ml) and pyridine (3 ml) overnight at room temperature. The reaction was dissolved in petroleum spirit (20 ml) and washed twice with saturated aqueous NaCl, once with saturated aqueous NaCl containing enough 4N sulfuric acid to render it just acidic and once with saturated aqueous NaCl. The solution was dried over anhydrous $MgSO_4$, and solvents were removed on a rotary evaporator. The residue (2.8 g) was chromatographed on silica gel (70 g) with 20% diethyl ether in petroleum spirit. Fractions containing product by GC-FID analysis were combined, solvents were removed on a rotary evaporator and the residue was distilled in a kugelrohr apparatus (100 °C/0.04 mm Hg) to give 2,8-diacetoxynonane (1.6 g, 6.6 mM).

The mass spectrum is shown in Fig. S2. ^{1}H NMR ($CDCl_3$, 400 MHz) δ 4.87 (q of t, J = 6,4, 7.2 Hz; 2H), 2.01 (s; 6H), 1.6-1.4 (m; 4H), 1.28 (m, 6H), 1.19 (d, J = 6.4 Hz; 6H). ^{13}C NMR ($CDCl_3$, 105 MHz) δ 170.79, 70.96, 35.85, 29.29, 25.32, 21.40, 19.97.

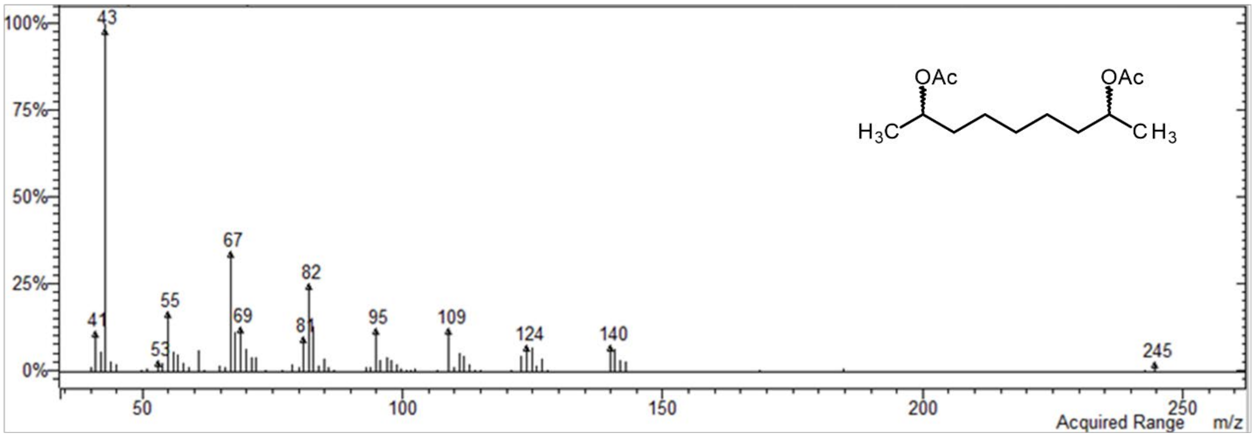

Fig. S2 Mass spectrum of 2,8-diacetoxynonane

Synthesis of 3-Acetoxynonane

3-Nonanol (0.72 g, 5 mM) was acetylated with acetic anhydride (0.75 ml) in pyridine (0.75 ml) at room temperature overnight. The mixture was dissolved in petroleum spirit (20 ml) and extracted twice with aqueous saturated NaCl solution and once with aqueous saturated NaCl with a few drops of 4N H_2SO_4 such that it was just acidic. The organic extracts were dried with anhydrous $MgSO_4$, filtered through silica gel (2 g), solvents removed on a rotary evaporator and the residue distilled in a kugelrohr apparatus (130 °C/10 mm Hg) to give 3-acetoxynonane (0.91 g, 98%). The mass spectrum is shown in Fig. S3.

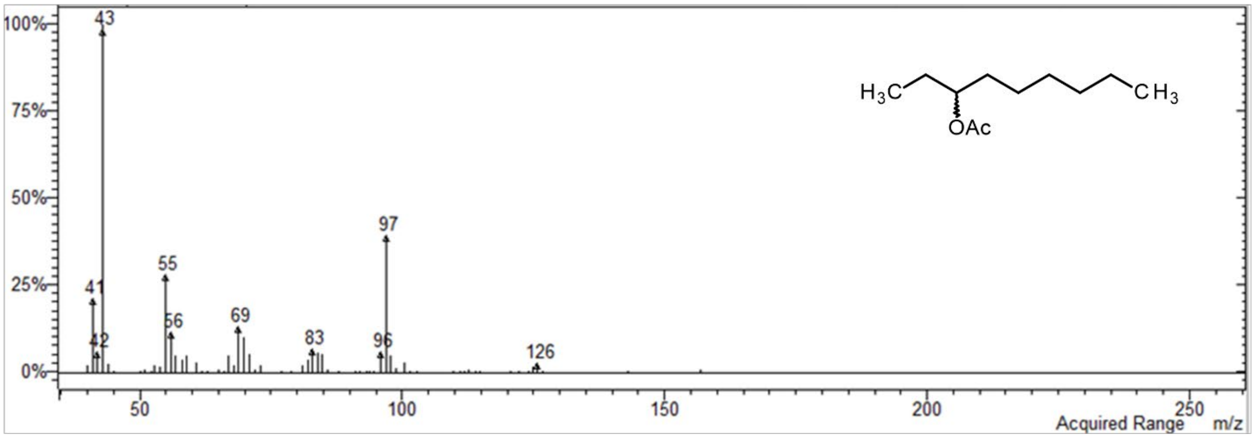

Fig. S3 Mass spectrum of 3-acetoxynonane

Table S1 Retention indices (RI) relative to retention times of *n*-alkanes of compounds in GC-MS analyses on non-polar VF5 and polar DBWax GC columns

	RI	
Compound	VF5	DBWax
Major female-specific compound	1533	1968
Minor female-specific compound	1235	1443
2,7-Diacetoxynonane	1529	1965/1968
2-Acetoxynonane	1234	1443
2,8-Diacetoxynonane	1551	2007
3-Acetoxynonane	1219	1416
